
JOURNAL INFORMATION
==============================
NLM Title Abbreviation: ScientificWorldJournal
Journal ID: ScientificWorldJournal
NLM Journal ID: 101131163
Journal ID: TSWJ

The Scientific World Journal

ISSN: 2356-6140
EISSN: 1537-744X
Publisher: Wiley

ARTICLE INFORMATION
==============================
PMCID: PMC6084736
PMID: 30147522
DOI: 10.1100/tsw.2001.187
Article ID: 762864
Article version: 1
Subjects: Short Report

Gene Expression is Selectively Affected along the Epididymis after Orchidectomy

Ezer Nadine N.
Robaire Bernard *
Departments of Pharmacology & Therapeutics and of Obstetrics and Gynecology, McGill University, 3655 Promenade Sir-William-Osler, Montreal, QC, Canada
*Bernard Robaire: brobaire@pharma.mcgill.ca
Publication date: 2001
Electronic publication date: 2001 Dec 4
Volume: 1
First page: 56
Last page: 56
Copyright: Copyright © 2001 Nadine N. Ezer and Bernard Robaire.
Copyright year: 2001
License: This is an open access article distributed under the Creative Commons Attribution License, which permits unrestricted use, distribution, and reproduction in any medium, provided the original work is properly cited.
License URL: https://creativecommons.org/licenses/by/3.0/

Funding: National Institutes of Health AG08321

==============================
Miami *Nature Biotechnology* Short Reports
*TheScientificWorld* (2001) 1 (S3), 56SR
ISSN 1532-2246; DOI 10.1100/tsw.2001.187

### GENE EXPRESSION IS SELECTIVELY AFFECTED ALONG THE EPIDIDYMIS AFTER ORCHIDECTOMY

Nadine N. Ezer and Bernard Robaire*

Departments of Pharmacology & Therapeutics and of Obstetrics and Gynecology, McGill University, 3655 Promenade Sir-William-Osler, Montreal, QC, Canada
* brobaire@pharma.mcgill.ca

**INTRODUCTION.** The epididymis is the site where immature spermatozoa from the testis acquire motility and the potential for fertilization. Although androgens decline more than 90% within 12 hours after orchidectomy, there is a wave-like pattern of epithelial principal cell apoptosis along the epididymis that progresses over 7 days (1); this coincides with the clearance of luminal fluid from the epididymal duct. The apoptotic response, detected by the TUNEL method, shows a peak at 2-3 days in the caput epididymidis and at 6 days in the cauda epididymidis. We hypothesize that orchidectomy has a dual effect on gene expression in epididymal cells; the first response is to the decreased serum androgen concentration while the second is to alter the content of the epididymal lumen.

**METHODS**. Male Brown Norway rats were bilaterally orchidectomized and either sacrificed immediately or 2 days later. Epididymides were removed, sectioned and flash-frozen at –80. RNA was extracted from the caput and cauda epididymal segments, reverse transcribed, and probed on cDNA arrays (Clontech$^{TM}$ Rat Stress Toxicology II arrays, n=3 arrays/treatment group/time point). Each array contained 475 rat cDNAs related to the stress-toxicology gene families in addition to control cDNAs. Arrays were analyzed using GeneSpring$^{TM}$ software. Only genes having twice background levels were considered to be expressed; changes in gene expression of at least twofold will be discussed.

**RESULTS.** On arrays using RNA from the control caput epididymidis, 195 genes were detected. Following orchidectomy, 73 genes are selectively up-regulated while only 6 genes were selectively down-regulated. Of those that increase, 6 are apoptosis-related including caspase-3, 5 are related to xenobiotic metabolism. Five genes are heat shock proteins, 12 are related to general cellular metabolism and 12 are related to transcription. Of the 6 genes that decrease, no single family of genes had more than 2 responders.

The response to orchidectomy in the cauda epididymidis differs remarkably from that of the caput epididymidis. Of the 256 genes expressed in control, only 12 genes are up-regulated after orchidectomy; only one of these, clusterin, is apoptosis-related. The major trend after orchidectomy is toward a decrease in gene expression, with 103 genes being selectively down-regulated. Of these genes, 3 are cell-cell adhesion receptors, 6 are related to xenobiotic metabolism, 29 are related to cellular metabolism and 9 are related to transcription. None of the genes down-regulated are related to the apoptosis.

**DISCUSSION.** The overall up-regulation of genes at 2 days post-orchidectomy in the caput epididymidis, particularly apoptotic, stress and transcription related genes, is in accordance with an active tissue response related to ongoing apoptotic changes. In the cauda, which has not yet been cleared of luminal fluid at 2 days, down-regulation of several metabolic, transcriptional and cell adhesion genes is likely the result of the stress induced by androgen-withdrawal alone. This is in accordance with androgen-withdrawal causing a general decline in overall cellular activity.

Based on gene expression changes reported here, we conclude that there is a differential response of genes in the epididymis between the withdrawal of circulating androgens and epididymal luminal content.

**ACKNOWLEDGEMENT.** Supported by NIH AG08321.

**REFERENCE.**

Fan X. and Robaire, B. (1998) Endocrinology 139(4), 2128-2136
